# Static and Free Vibration Analyses of Single-Walled Carbon Nanotube (SWCNT)–Substrate Medium Systems

**DOI:** 10.3390/nano12101740

**Published:** 2022-05-19

**Authors:** Suchart Limkatanyu, Worathep Sae-Long, Hamid Mohammad-Sedighi, Jaroon Rungamornrat, Piti Sukontasukkul, Thanongsak Imjai, Hexin Zhang

**Affiliations:** 1 Department of Civil and Environmental Engineering, Faculty of Engineering, Prince of Songkla University, Songkhla 90112, Thailand; suchart.l@psu.ac.th; 2 Civil Engineering Program, School of Engineering, University of Phayao, Phayao 56000, Thailand; 3 Mechanical Engineering Department, Faculty of Engineering, Shahid Chamran University of Ahvaz, Ahvaz 6135783151, Iran; hmsedighi@gmail.com; 4 Drilling Center of Excellence and Research Center, Shahid Chamran University of Ahvaz, Ahvaz 6135783151, Iran; 5 Applied Mechanics and Structures Research Unit, Department of Civil Engineering, Faculty of Engineering, Chulalongkorn University, Bangkok 10330, Thailand; jaroon.r@chula.ac.th; 6 Construction and Building Materials Research Center, Department of Civil Engineering, King Mongkut’s University of Technology North Bangkok, Bangkok 10800, Thailand; piti.s@eng.kmutnb.ac.th; 7 School of Engineering and Technology, Center of Excellence in Sustainable Disaster Management, Walailak University, Nakhon Si Thammarat 80161, Thailand; thanongsak.im@wu.ac.th; 8 School of Engineering and the Built Environment, Edinburgh Napier University, Edinburgh EH14 1DJ, UK; j.zhang@napier.ac.uk

**Keywords:** free vibration, nanobar, modified strain-gradient theory, surface elasticity theory, elastic substrate media, Hamilton’s principle

## Abstract

This paper proposes a novel nanobar–substrate medium model for static and free vibration analyses of single-walled carbon nanotube (SWCNT) systems embedded in the elastic substrate medium. The modified strain-gradient elasticity theory is utilized to account for the material small-scale effect, while the Gurtin–Murdoch surface theory is employed to represent the surface energy effect. The Winkler foundation model is assigned to consider the interactive mechanism between the nanobar and its surrounding substrate medium. Hamilton’s principle is used to consistently derive the system governing equation, initial conditions, and classical as well as non-classical boundary conditions. Two numerical simulations are employed to demonstrate the essence of the material small-scale effect, the surface energy effect, and the surrounding substrate medium on static and free vibration responses of single-walled carbon nanotube (SWCNT)–substrate medium systems. The simulation results show that the material small-scale effect, the surface energy effect, and the interaction between the substrate and the structure led to a system-stiffness enhancement both in static and free vibration analyses.

## 1. Introduction

Over the last few decades, nano-sized structures have found a wide spectrum of applications in nano-sized devices due to their superior and unique mechanical properties [1,2,3,4,5], for example, nanofilms [6], nanobiosensors [7], nanomotors [8], nanoswitches [9], nano-electro-mechanical systems (NEMS) [10], nanowires [11], and atomic force microscopes (AFM) [12]. To reliably design and fabricate such tiny devices, mechanical characteristics of their small-sized structural components are utterly crucial and necessary. Generally, the most straightforward way to characterize the mechanical properties of structures is the experimental approach. Unfortunately, conducting an experiment on nano-sized structures is extremely cumbersome and requires a high-precision testing apparatus and a unique testing procedure [13,14,15]. The atomistic modeling approach based on the quantum mechanics theory could be a viable choice to characterize the mechanical properties of small-sized structures, but high computational costs and complex computational procedures inherently arise with this modeling approach [16,17,18,19,20]. Therefore, the atomistic modeling approach is valid only to systems with small numbers of molecules and atoms. To remedy the aforementioned difficulties inherent to experimental and atomistic modeling approaches, there is a pressing need for a more efficient modeling approach to characterize the mechanical properties of nano-sized structures. Such a modeling approach could be possible with unification of enhanced elasticity theories and structural mechanics models, thus leading to the so-called “*enhanced*” structural mechanics models. A family of enhanced structural mechanics models has been formulated by researchers worldwide and ranges from a relatively simple bar model to a sophisticated curvilinear shell model [21,22,23,24,25]. Small-scale and/or size-dependent effects have been incorporated into these enhanced structural mechanics models. The small-scale effect is induced by the long-range inter-atomic interaction, while the size-dependent effect is caused by excessive energy of atoms at the free surface.

When the structural dimension is in the order of nanometers, the discrete nature of materials becomes essential and induces the small-scale effect. In the research community, several enhanced elasticity theories have been formulated by several researchers to consider the small-scale effect, such as nonlocal elasticity theory [26,27], strain-gradient theory [28], modified strain-gradient elasticity theory [13], couple stress theory [29], and modified couple stress theory [30]. It is worth mentioning that the common feature of these enhanced elasticity theories is the supplement of material length-scale parameters to account for the small-scale effect associated with the discrete nature of materials. As the most widely used among these enhanced elasticity theories, the nonlocal elasticity theory of Eringen [26,27] has been employed to characterize the small-scale effect on static and dynamic responses of nano-sized structures for the last two decades [31,32,33,34,35,36,37,38,39]. For example, Reddy and Pang [31] developed the Euler–Bernoulli and Timoshenko beam models based on the Eringen nonlocal differential constitutive relation to study the effect of the nonlocal parameter on the bending, buckling, and natural vibration of straight beams. Wang et al. [32] investigated the transverse shear deformation within the micro- and nano-beams by using the Timoshenko beam model based on Eringen’s nonlocal elasticity theory. Juntarasaid et al. [33] extended the Euler–Bernoulli beam model of Reddy and Pang [31] to consider the surface effects for bending and buckling analysis of the nanowire. Barretta et al. [34] developed the Eringen differential law by the suggestion of an additional term involving the derivative of the axial stress. Ebrahimi and Shafiei [35] proposed a rotating, functionally graded (FG) Timoshenko nanobeam based on Eringen’s nonlocal theory to study the size-dependent vibration behavior. Limkatanyu et al. [36] employed the so-called “*strain-driven*” nonlocal elasticity model as proposed by Eringen [27] to enrich the Euler–Bernoulli beam model. Next, Nguyen et al. [37] studied the bending, buckling, and post-buckling of nanobeams undergoing large displacements and rotations based on the Eringen nonlocal beam model. Subsequently, Sayyad and Ghugal [38] presented a theoretical unification of twenty-one nonlocal beam theories based on Eringen’s nonlocal elasticity theory for the bending, buckling, and vibration analysis of FG nanobeams, while Lal and Dangi [39] proposed the nonlocal Timoshenko beam model for vibration analysis of bi-directional FG moderately thick nanobeams. However, skeptical and inconsistent responses could arise with the nonlocal elasticity model [40]. For example, Peddieson et al. [40] demonstrated that the beam model based on the Eringen nonlocal differential form presents no small-scale effect on the displacement response of the cantilever beam under an end load. This peculiar response was subsequently defined as a “*paradox*” [40,41]. It was diagnosed by Romano et al. [41] that adoption of the Eringen nonlocal differential model can result in an ill-posed structural mechanics problem (bounded domain). Furthermore, Koutsoumaris et al. [42] showed that the quadratic energy functional form of elasticity does not exist for the Eringen nonlocal differential model, and Ma et al. [43] pointed out that there is ambiguity in the conjugate stress–strain pairs in this nonlocal constitutive model. To deviate from skeptical and inconsistent features inherent to the Eringen nonlocal differential model, several researchers have paid attention to strain-gradient-type constitutive models. Within the framework of the strain-gradient-type constitutive model, there is no ambiguity in the conjugate stress–strain pairs and there exists the quadratic energy functional form of elasticity, thus leading to a rational nano-sized structural model. Among a family of strain-gradient-type constitutive models, the modified strain-gradient elasticity model proposed by Lam et al. [13] is of particular interest in the present work since strain-gradient measures associated with different deformations are naturally considered in this strain-gradient-type model, as will be pursued herein.

Besides the abovementioned small-scale effect, the size-dependent characteristic induced by the surface energy effect becomes essential when the structural dimension is in the order of nanometers. The surface energy effect is related to excessive energy stored in the bulk surface of nano-sized structures. Both atomistic modeling and experimental studies on the nano-sized structure have confirmed the existence of the surface energy effect [44,45,46]. To incorporate the surface energy effect into the structural mechanics model, several researchers have used the surface elasticity theory proposed by Gurtin and Murdoch [47,48]. The fundamental assumption of this surface theory is that the wrapping surface layer is considered as a zero-thickness, two-dimensional membrane perfectly bonded to its embracing bulk material and possessing its own elastic properties.

In nanomaterials and nanodevices, nanobars have often been assembled into larger parts through elastic substrate media [49]. Therefore, the interactive mechanism between the bar and its surrounding substrate medium is paramount to the design and control of the performance of such materials and devices during their lifetime services. Up to now, nanobar–substrate medium models available in the literature have mainly been developed for static analyses of nanobar–substrate medium systems under tensile loadings. For example, Limkatanyu et al. [50,51] developed the bar–substrate medium model based on the nonlocal elasticity theory of Eringen [26,27] to characterize axial responses of nanowire-substrate medium systems. Sae-Long et al. [52] and Limkatanyu et al. [53] unified the thermodynamic-based strain-gradient model of Barretta and Marotti de Sciarra [54] and the surface elasticity model of Gurtin and Murdoch [47,48] to develop a “*paradox-free*” nanobar-elastic substrate medium model. Sae-Long et al. [55] combined the four-order strain-gradient model of Narendar and Gopalakrishnan [56] with the surface elasticity model of Gurtin and Murdoch [47,48] to formulate the sixth-order bar-elastic substrate medium model containing one material length-scale parameter. Apart from the static characteristic of nanobar–substrate medium systems, their dynamic characteristic is also essential to rational design and fabrication processes of nanomaterials and nanodevices. Consequently, there is still room to add a more general mathematical model of nanobar–substrate medium systems into the research community. To the authors’ best knowledge, the present study proposes, for the first time, a combination of the modified strain-gradient elasticity model of Lam et al. [13] and the surface elasticity model of Gurtin and Murdoch [47,48] to formulate a rational nanobar–substrate medium model for static and dynamic analyses.

The content of the present study is as follows: First, the interactive mechanism between the bar and its surrounding substrate medium is presented. Then, the modified strain-gradient elasticity model of Lam et al. [13] and the surface elasticity model of Gurtin and Murdoch [47,48] are discussed. Next, Hamilton’s principle is employed to reveal the system governing equation, initial conditions, and classical as well as non-classical boundary conditions. These equations establish a complete set of the mathematical tool to perform static and free vibration analyses of the nanobar–substrate medium systems. Finally, two numerical simulations are employed to demonstrate the essence of the material small-scale effect, the surface energy effect, and the surrounding substrate medium on static and free vibration responses of single-walled carbon nanotube (SWCNT)–substrate medium systems.

## 2. Nanobar–Substrate Medium Interaction

**Figure 1:**
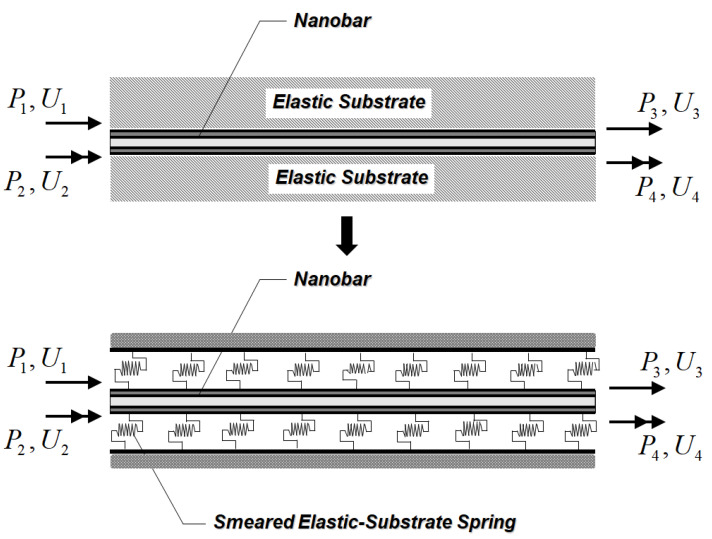
Nanobar–substrate medium system.

In the present study, the interactive mechanism between the nanobar and its surrounding substrate medium is characterized by the Winkler foundation model [57]. Based on this foundation model, the surrounding substrate medium is considered as smeared tangential springs distributed along the nanobar length, as shown in Figure 1. The constitutive relation between the substrate interactive force, $D_{s}\left(x\right)$, and the substrate deformation, $\Delta_{s}\left(x\right)$, is [57]:

(1) $$D_{s}\left(x\right)=k_{s}\Delta_{s}\left(x\right)$$

where $k_{s}$ is the substrate medium stiffness.

## 3. Modified Strain-Gradient Theory

The modified strain-gradient theory employed herein is one of simplified variants of Mindlin’s strain-gradient theory [28,58] and was first proposed by Lam et al. [13]. To consider the material small-scale effect inherent to nano-sized structures, the strain-gradient theory asserts that the stress at a generic point is described as a function of local strain and strain gradients at that point. Consequently, the modified strain-gradient theory of Lam et al. [13] possesses three more material constants related to strain gradients in addition to two conventional material constants (Lame constants).

For a modified strain-gradient elastic body, the stored elastic strain energy, U, is given by Lam et al. [13] as:

(2) $$U=\frac{1}{2}\int_{V}\left(\sigma_{ij}\varepsilon_{ij}+p_{i}\gamma_{i}+\tau_{ijk}^{\left(1\right)}\eta_{ijk}^{\left(1\right)}+m_{ij}^{s}\chi_{ij}^{s}\right)\;dV$$

where V represents the volume of the elastic body, $\varepsilon_{ij}$ represents the infinitesimal strain and is the conjugate–work pair of the classical stress, $\sigma_{ij}$, $\gamma_{i}$, $\eta_{ijk}^{\left(1\right)}$, and $\chi_{ij}^{s}$ represent strain-gradient measures related to the dilatation gradient, the deviatoric stretch gradient, and the symmetric rotation gradient, respectively, and $p_{i}$, $\tau_{ijk}^{\left(1\right)}$, and $m_{ij}^{s}$ represent the higher-order stresses and are the conjugate–work pairs of the strain-gradient measures $\gamma_{i}$, $\eta_{ijk}^{\left(1\right)}$, and $\chi_{ij}^{s}$, respectively.

In the modified strain-gradient theory, the constitutive relations between stress and strain quantities are [59]:

(3) $$\sigma_{ij}^{}=\lambda \delta_{ij}^{}\varepsilon_{mm}^{}+2\mu \varepsilon_{ij}^{}$$

(4) $$p_{i}^{}=2\mu l_{0}^{2}\gamma_{i}^{}$$

(5) $$\tau_{ijk}^{\left(1\right)}=2\mu l_{1}^{2}\eta_{ijk}^{\left(1\right)}$$

(6) $$m_{ij}^{s}=2\mu l_{2}^{2}\chi_{ij}^{s}$$

where λ and μ are Lame constants, $l_{0}$, $l_{1}$, and $l_{2}$ are the material length-scale parameters related to the dilatation gradient, the deviatoric stretch gradient, and the symmetric rotation gradient, respectively, and $\delta_{ij}^{}$ is Kronecker delta.

The strain, $\varepsilon_{ij}$, and the strain-gradient measures $\gamma_{i}$, $\eta_{ijk}^{\left(1\right)}$, and $\chi_{ij}^{s}$ are defined as [59]:

(7) $$\varepsilon_{ij}^{}=\frac{1}{2}\left(u_{i,j}^{}+u_{j,i}^{}\right)$$

(8) $$\gamma_{i}^{}=\varepsilon_{mm,i}^{}$$

(9) $$\begin{array}{l} \eta_{ijk}^{\left(1\right)}=\frac{1}{3}\left(\varepsilon_{jk,i}^{}+\varepsilon_{ki,j}^{}+\varepsilon_{ij,k}^{}\right)-\frac{1}{15}[\delta_{ij}^{}\left(\varepsilon_{mm,k}^{}+2\varepsilon_{mk,m}^{}\right)\\ \;\;\;\;\;\;\;\;+\delta_{jk}^{}\left(\varepsilon_{mm,i}^{}+2\varepsilon_{mi,m}^{}\right)+\delta_{ki}^{}\left(\varepsilon_{mm,j}^{}+2\varepsilon_{mj,m}^{}\right)] \end{array}$$

(10) $$\chi_{ij}^{s}=\frac{1}{2}\left(e_{ipq}\varepsilon_{qj,p}+e_{jpq}\varepsilon_{qi,p}\right)$$

where $u_{i}^{}$ is the displacement vector and $e_{ijk}^{}$ is the permutation symbol.

## 4. Surface Elasticity Theory

**Figure 2:**
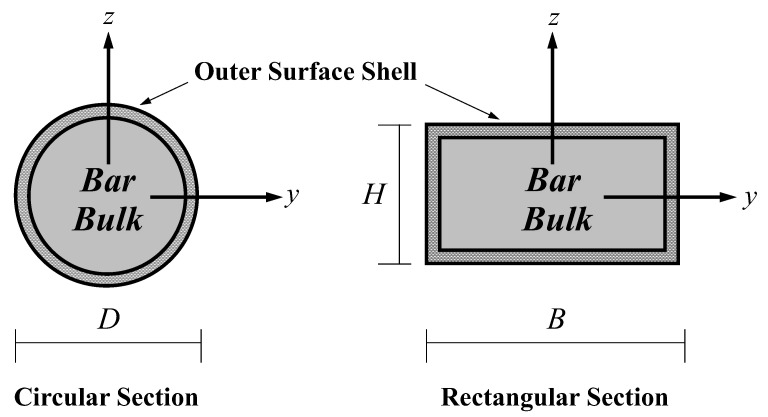
Composite nanobar cross-section: bar-bulk and outer wrapping surface shell.

As the size of a structure is in the range of nanometers, the free energy at the surface of a bulk material becomes comparable to the energy stored in the bulk material. Therefore, the size-dependent phenomenon induced by this surface-free energy is essential in characterizing the mechanical response of nano-sized structures. To incorporate this size-dependent phenomenon into the proposed nanobar–substrate medium model, the present study employs the surface elasticity theory proposed by Gurtin and Murdoch [47,48]. Based on the hypothesis of this surface elasticity model, the nanobar cross-section is assumed to consist of a solid core and its wrapping outer surface shell, as shown in Figure 2. The solid core and its wrapping outer surface shell are assumed to be in a perfect bond condition. The wrapping outer surface shell is taken as a mathematically zero-thickness elastic layer. The constitutive relation of the surface layer can be expressed as [47,48]:

(11) $$\tau_{\alpha \beta }^{sur}=\left[\tau_{0}^{sur}+\left(\lambda_{0}^{sur}+\mu_{0}^{sur}\right)u_{\gamma ,\gamma }^{sur}\right]\delta_{\alpha \beta }+\mu_{0}^{sur}\left(u_{\alpha ,\beta }^{sur}+u_{\beta ,\alpha }^{sur}\right)-\tau_{0}^{sur}u_{\beta ,\alpha }^{sur}$$

where $\lambda_{0}^{sur}$ and $\mu_{0}^{sur}$ are the surface elastic constants, $\tau_{\alpha \beta }^{sur}$ is the in-plane components of the surface stress, $\tau_{0}^{sur}$ is the residual surface stress based on the unconstrained conditions and is determined from the atomistic simulation [45], and $u_{}^{sur}$ is the surface layer deformation.

## 5. Nanobar–Substrate Medium Model: Single-Walled Carbon Nanotube (SWCNT)–Substrate Medium System

### 5.1. Kinematics

**Figure 3:**
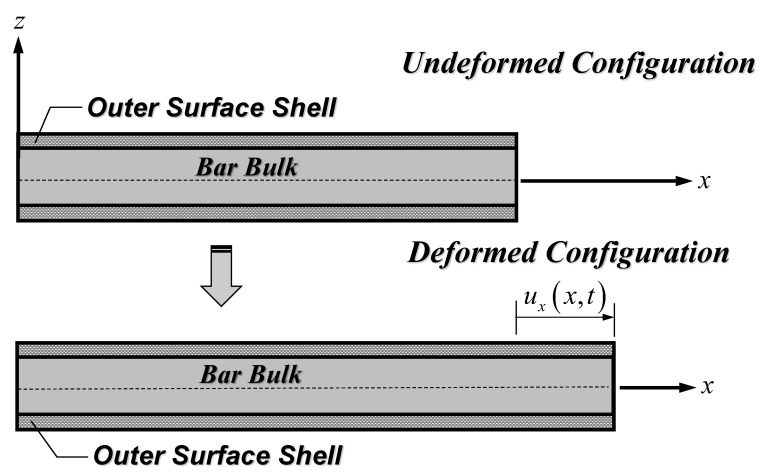
Nanobar kinematical description.

In the present work, a SWCNT is considered as a bar, thus focusing merely on the axial response. Therefore, only the bar axial displacement, $u_{x}\left(x,t\right)$, shown in Figure 3 is relevant. It is worth mentioning that the bar axial displacement, $u_{x}\left(x,t\right)$, is described as a function of the spatial variable x and the temporal variable t, since both static and free vibration responses of SWCNT–substrate medium systems are investigated in this study.

Following the bar kinematics of Figure 3, the non-zero strain of Equation (7) and strain-gradient measures of Equations (8)–(10) are [59]:

(12) $$\varepsilon_{xx}^{}=\frac{\partial u_{x}\left(x,t\right)}{\partial x}$$

(13) $$\gamma_{x}^{}=\frac{\partial \varepsilon_{xx}^{}}{\partial x}=\frac{\partial^{2}u_{x}\left(x,t\right)}{\partial x^{2}}$$

(14) $$\eta_{xxx}^{\left(1\right)}=\frac{2}{5}\frac{\partial \varepsilon_{xx}}{\partial x}=\frac{2}{5}\frac{\partial^{2}u_{x}\left(x,t\right)}{\partial x^{2}}$$

(15) $$\eta_{xyy}^{\left(1\right)}=\eta_{xzz}^{\left(1\right)}=\eta_{yxy}^{\left(1\right)}=\eta_{yyx}^{\left(1\right)}=\eta_{zxz}^{\left(1\right)}=\eta_{zzx}^{\left(1\right)}=-\frac{1}{5}\frac{\partial \varepsilon_{xx}}{\partial x}=-\frac{1}{5}\frac{\partial^{2}u_{x}\left(x,t\right)}{\partial x^{2}}$$

It is noted that the bar kinematics of Figure 3 results in a vanishing symmetric rotation gradient, $\chi_{ij}^{s}$. Consequently, the higher-order stress, $m_{ij}^{s}$, also vanishes based on the constitutive relation of Equation (6), and the material length-scale parameter, $l_{2}$, becomes unnecessary.

Employing Equations (12)–(15), the non-vanishing stress quantities (classical and higher-order stresses) of Equations (3)–(5) can be expressed in terms of the bar axial displacement, $u_{x}\left(x,t\right)$, as [59]:

(16) $$\sigma_{xx}^{}=E\frac{\partial u_{x}\left(x,t\right)}{\partial x}$$

(17) $$p_{x}^{}=2\mu l_{0}^{2}\frac{\partial^{2}u_{x}\left(x,t\right)}{\partial x^{2}}$$

(18) $$\tau_{xxx}^{\left(1\right)}=\frac{4}{5}\mu l_{1}^{2}\frac{\partial^{2}u_{x}\left(x,t\right)}{\partial x^{2}}$$

(19) $$\tau_{xyy}^{\left(1\right)}=\tau_{xzz}^{\left(1\right)}=\tau_{yxy}^{\left(1\right)}=\tau_{yyx}^{\left(1\right)}=\tau_{zxz}^{\left(1\right)}=\tau_{zzx}^{\left(1\right)}=-\frac{2}{5}\mu l_{1}^{2}\frac{\partial^{2}u_{x}\left(x,t\right)}{\partial x^{2}}$$

with E being the elastic modulus of the bar-bulk material. It is worth mentioning that the effect of Poisson’s ratio, ν, is neglected in the present study. This assumption was also made for the modified strain-gradient bar model proposed by Akgöz and Civalek [59].

Enforcing the full compatibility condition of the nanobar–substrate medium system ($\Delta_{s}\left(x,t\right)=u_{x}\left(x,t\right)$), the substrate medium constitutive relation of Equation (1) can be expressed in terms of the bar axial displacement, $u_{x}\left(x,t\right)$, as [57]:

(20) $$D_{s}\left(x,t\right)=k_{s}\Delta_{s}^{}\left(x,t\right)=k_{s}u_{x}\left(x,t\right)$$

Following the full compatibility condition of the bar-bulk-wrapping surface layer system ($\varepsilon_{xx}^{sur}\left(x,t\right)=\varepsilon_{xx}\left(x,t\right)$) and the bar section kinematics, the surface layer constitutive relation of Equation (11) can be written in terms of the bar axial displacement, $u_{x}\left(x,t\right)$, as [47,48]:

(21) $$\tau_{xx}^{sur}\left(x,t\right)-\tau_{0}^{sur}=E_{}^{sur}\varepsilon_{xx}^{sur}\left(x,t\right)=E_{}^{sur}\frac{\partial u_{x}\left(x,t\right)}{\partial x}$$

where $\tau_{xx}^{sur}\left(x,t\right)$ and $\tau_{0}^{sur}$ are, respectively, the surface stress and the residual surface stress in the axial direction, $E_{}^{sur}=\lambda_{0}^{sur}+2\mu_{0}^{sur}$ is the surface elastic modulus, and $\varepsilon_{xx}^{sur}\left(x,t\right)$ is the surface layer strain.

### 5.2. Formulation: Hamilton’s Principle

Based on the system constitutive relations of Equations (16)–(21), the elastic strain energy, U, stored in the nanobar–substrate system can be expressed in terms of the bar axial displacement, $u_{x}\left(x,t\right)$, as:

(22) $$\begin{array}{l} U=\underset{\mathrm{Nanobar}\;\;\mathrm{contribution}}{\underset{︸}{\overset{\mathrm{Local}\;\;\mathrm{term}}{\overset{︷}{\frac{1}{2}{\int_{0}^{L}EA\left(\frac{\partial u_{x}\left(x,t\right)}{\partial x}\right)}^{2}dx}}}}+\underset{\mathrm{Surface}-\mathrm{energy}\;\;\mathrm{contribution}}{\underset{︸}{\frac{1}{2}{\int_{0}^{L}E_{}^{sur}\Gamma \left(\frac{\partial u_{x}\left(x,t\right)}{\partial x}\right)}^{2}dx}}\\ \;\;\;\;\;+\underset{\mathrm{Nanobar}\;\;\mathrm{contribution}}{\underset{︸}{\overset{\mathrm{Dilatation}\;\;\mathrm{gradient}\;\;\mathrm{term}}{\overset{︷}{\frac{1}{2}{\int_{0}^{L}2\mu Al_{0}^{2}\left(\frac{\partial^{2}u_{x}\left(x,t\right)}{\partial x^{2}}\right)}^{2}dx}}+\overset{\mathrm{Deviatoric}\;\;\mathrm{stretch}\;\;\mathrm{gradient}\;\;\mathrm{term}\;\;}{\overset{︷}{\frac{1}{2}{\int_{0}^{L}\frac{4}{5}\mu Al_{1}^{2}\left(\frac{\partial^{2}u_{x}\left(x,t\right)}{\partial x^{2}}\right)}^{2}dx}}}}\\ \;\;\;\;\;+\underset{\mathrm{Substrate}\;-\mathrm{medium}\;\;\mathrm{contribution}}{\underset{︸}{\frac{1}{2}\int_{0}^{L}k_{s}{\left(u_{x}\left(x,t\right)\right)}^{2}dx}} \end{array}$$

where $A=\int_{A}\;dA$ is the nanobar sectional area, and $\Gamma =\oint_{\Gamma }d\Gamma$ is the nanobar sectional perimeter.

The first variation of the elastic strain energy, U, of Equation (22) during the time interval [$t_{0}$, $t_{1}$] is:

(23) $$\begin{array}{l} \delta U=\int_{t_{0}}^{t_{1}}[\int_{0}^{L}EA\left(\frac{\partial u_{x}\left(x,t\right)}{\partial x}\right)\left(\frac{\partial \delta u_{x}\left(x,t\right)}{\partial x}\right)dx+\int_{0}^{L}E_{}^{sur}\Gamma \left(\frac{\partial u_{x}\left(x,t\right)}{\partial x}\right)\left(\frac{\partial \delta u_{x}\left(x,t\right)}{\partial x}\right)dx\\ \;\;\;\;\;\;\;\;\;+\int_{0}^{L}2\mu Al_{0}^{2}\left(\frac{\partial^{2}u_{x}\left(x,t\right)}{\partial x^{2}}\right)\left(\frac{\partial^{2}\delta u_{x}\left(x,t\right)}{\partial x^{2}}\right)dx+\int_{0}^{L}\frac{4}{5}\mu Al_{1}^{2}\left(\frac{\partial^{2}u_{x}\left(x,t\right)}{\partial x^{2}}\right)\left(\frac{\partial^{2}\delta u_{x}\left(x,t\right)}{\partial x^{2}}\right)dx\\ \;\;\;\;\;\;\;\;\;+\int_{0}^{L}k_{s}\left(u_{x}\left(x,t\right)\right)\left(\delta u_{x}\left(x,t\right)\right)\;dx]dt \end{array}$$

In order to remove all differential operations from the virtual axial displacement, $\delta u_{x}\left(x,t\right)$, integration by parts is applied to Equation (23), thus resulting in the following expression:

(24) $$\begin{array}{l} \delta U=\int_{t_{0}}^{t_{1}}\int_{0}^{L}\delta u_{x}\left(x,t\right)\left[{\left(EA\right)}_{}^{H}\frac{\partial^{4}u_{x}\left(x,t\right)}{\partial x^{4}}-{\left(EA\right)}_{}^{L}\frac{\partial^{2}u_{x}\left(x,t\right)}{\partial x^{2}}+k_{s}u_{x}\left(x,t\right)\right]\;dx\;dt\\ \;\;\;\;\;\;\;\;+{\int_{t_{0}}^{t_{1}}\delta u_{x}\left(x,t\right)\left[-{\left(EA\right)}_{}^{H}\frac{\partial^{3}u_{x}\left(x,t\right)}{\partial x^{3}}+{\left(EA\right)}_{}^{L}\frac{\partial u_{x}\left(x,t\right)}{\partial x}\right]}_{x=0}^{x=L}dt\\ \;\;\;\;\;\;\;\;+{\int_{t_{0}}^{t_{1}}\frac{\partial \delta u_{x}\left(x,t\right)}{\partial x}\left[{\left(EA\right)}_{}^{H}\frac{\partial^{2}u_{x}\left(x,t\right)}{\partial x^{2}}\right]}_{x=0}^{x=L}dt \end{array}$$

where ${\left(EA\right)}_{}^{H}=2\mu Al_{0}^{2}+\frac{4}{5}\mu Al_{1}^{2}$ represents the higher-order axial stiffness considering the bar-bulk dilatation strain gradient and the bar-bulk deviatoric stretch strain gradient, and ${\left(EA\right)}_{}^{L}=EA+E_{}^{sur}\Gamma$ is the lower-order axial stiffness combining the bar-bulk and surface layer contributions.

Next, the first variation of the external work, W, performed due to the axially distributed load, $q_{x}\left(x,\;t\right)$, and end forces, P, as shown in Figure 1, during the time interval [$t_{0}$, $t_{1}$] can be expressed as:

(25) $$\delta W=\int_{t_{0}}^{t_{1}}\int_{0}^{L}q_{x}\left(x,t\right)\delta u_{x}\left(x,t\right)\;dx\;dt+\delta U^{T}P$$

where the displacement vector $U={\left\{\begin{array}{cccc} U_{1} & U_{2} & U_{3} & U_{4} \end{array}\right\}}^{T}$ collects end displacements of the system, and the force vector $P={\left\{\begin{array}{cccc} P_{1} & P_{2} & P_{3} & P_{4} \end{array}\right\}}^{T}$ contains end forces of the system.

Finally, the first variation of the kinetic energy, T, for the axial response during the time interval [$t_{0}$, $t_{1}$] is given by Akgöz and Civalek [59] as:

(26) $$\delta K=-\int_{t_{0}}^{t_{1}}\int_{0}^{L}\rho A\frac{\partial^{2}u_{x}\left(x,t\right)}{\partial t^{2}}\delta u_{x}\left(x,t\right)\;dxdt+{\int_{0}^{L}\delta u_{x}\left(x,t\right)\left[\rho A\frac{\partial u_{x}\left(x,t\right)}{\partial t}\right]}_{t_{0}}^{t_{1}}\;dx$$

where ρ is the bar-bulk mass density.

Recalling Hamilton’s principle, the relations in Equations (24)–(26) can be written together as:

(27) $$\begin{array}{l} \delta \left\{\int_{t_{0}}^{t_{1}}\left[T-\left(U-W\right)\right]\;dt\right\}=0\\ =\int_{t_{0}}^{t_{1}}\int_{0}^{L}\delta u_{x}\left(x,t\right)[-{\left(EA\right)}_{}^{H}\frac{\partial^{4}u_{x}\left(x,t\right)}{\partial x^{4}}+{\left(EA\right)}_{}^{L}\frac{\partial^{2}u_{x}\left(x,t\right)}{\partial x^{2}}-k_{s}u_{x}\left(x,t\right)\\ +q_{x}\left(x,t\right)-\rho A\frac{\partial^{2}u_{x}\left(x,t\right)}{\partial t^{2}}]dxdt+\delta U^{T}P\\ +{\int_{t_{0}}^{t_{1}}\delta u_{x}\left(x,t\right)\left[{\left(EA\right)}_{}^{H}\frac{\partial^{3}u_{x}\left(x,t\right)}{\partial x^{3}}-{\left(EA\right)}_{}^{L}\frac{\partial u_{x}\left(x,t\right)}{\partial x}\right]}_{x=0}^{x=L}dt\\ +{\int_{t_{0}}^{t_{1}}\frac{\partial \delta u_{x}\left(x,t\right)}{\partial x}\left[-{\left(EA\right)}_{}^{H}\frac{\partial^{2}u_{x}\left(x,t\right)}{\partial x^{2}}\right]}_{x=0}^{x=L}dt\\ +{\int_{0}^{L}\delta u_{x}\left(x,t\right)\left[\rho A\frac{\partial u_{x}\left(x,t\right)}{\partial t}\right]}_{t_{0}}^{t_{1}}\;dx=0 \end{array}$$

Accounting for the arbitrariness of $\delta u_{x}\left(x,t\right)$, the governing differential equation of motion (Euler–Lagrange equation) for a nanobar–substrate medium system can be deduced from Equation (27) as:

(28) $$-{\left(EA\right)}_{}^{H}\frac{\partial^{4}u_{x}\left(x,t\right)}{\partial x^{4}}+{\left(EA\right)}_{}^{L}\frac{\partial^{2}u_{x}\left(x,t\right)}{\partial x^{2}}-k_{s}u_{x}\left(x,t\right)+q_{x}\left(x,t\right)=\rho A\frac{\partial^{2}u_{x}\left(x,t\right)}{\partial t^{2}}$$

It is worth remarking that Equation (28) becomes identical to the governing differential equation of motion for a modified strain-gradient nanobar model proposed by Akgöz and Civalek [59] when the surrounding substrate medium and the surface energy effect are neglected ($k_{s}=E^{sur}=\tau_{0}^{sur}=0$). Moreover, when the effects of the material small-scale and surface energy are all neglected ($l_{0}^{}=l_{1}=E^{sur}=\tau_{0}^{sur}=0$), Equation (28) degenerates to the governing differential equation of motion for a bar embedded in the Winkler foundation [51].

Considering the arbitrariness of $\delta u_{x}\left(x,t\right)$ and δU on boundary terms in Equation (27) yields initial conditions as well as boundary conditions of the nanobar–substrate medium system as:

Initial conditions:

(29) $$\delta u_{x}\left(x,t_{1}\right){\left[\rho A\frac{\partial u_{x}\left(x,t_{1}\right)}{\partial t}\right]}_{t=t_{1}}-\delta u_{x}\left(x,t_{0}\right){\left[\rho A\frac{\partial u_{x}\left(x,t_{0}\right)}{\partial t}\right]}_{t=t_{0}}=0$$

Boundary conditions:

(30) $$\begin{array}{l} P_{1}={\left[{\left(EA\right)}_{}^{H}\frac{\partial^{3}u_{x}\left(0,t\right)}{\partial x^{3}}-{\left(EA\right)}_{}^{L}\frac{\partial u_{x}\left(0,t\right)}{\partial x}\right]}_{x=0};\;P_{2}={\left[-{\left(EA\right)}_{}^{H}\frac{\partial^{2}u_{x}\left(0,t\right)}{\partial x^{2}}\right]}_{x=0}\\ P_{3}=-{\left[{\left(EA\right)}_{}^{H}\frac{\partial^{3}u_{x}\left(L,t\right)}{\partial x^{3}}-{\left(EA\right)}_{}^{L}\frac{\partial u_{x}\left(L,t\right)}{\partial x}\right]}_{x=L};\;P_{4}={\left[{\left(EA\right)}_{}^{H}\frac{\partial^{2}u_{x}\left(L,t\right)}{\partial x^{2}}\right]}_{x=L} \end{array}$$

### 5.3. Analytical Solution of the Nanobar–Substrate Medium System: Static Analysis

When the inertia force $\rho A\left(\partial^{2}u_{x}\left(x,t\right)/\partial t^{2}\right)$ on the right-hand side is omitted, Equation (28) degenerates to the governing differential equilibrium equation of a nanobar–substrate medium system:

(31) $${\left(EA\right)}_{}^{H}\frac{\partial^{4}u_{x}\left(x\right)}{\partial x^{4}}-{\left(EA\right)}_{}^{L}\frac{\partial^{2}u_{x}\left(x\right)}{\partial x^{2}}+k_{s}u_{x}\left(x\right)-q_{x}\left(x\right)=0$$

The general solution to the governing differential equilibrium equation of Equation (31) can be written as:

(32) $$u_{x}\left(x\right)=u_{x}^{h}\left(x\right)+u_{x}^{p}\left(x\right)$$

where $u_{x}^{h}\left(x\right)$ is the homogeneous solution for $q_{x}\left(x\right)=0$, and $u_{x}^{p}\left(x\right)$ is the particular solution and is dictated by the axially distributed load, $q_{x}\left(x\right)$.

It is observed that Equation (31) is in the same form as the governing differential equilibrium equation of the beam on the Winkler–Pasternak foundation [60]. Therefore, the homogeneous solution, $u_{x}^{h}\left(x\right)$, given by Limkatanyu et al. [60] can be employed for the present problem. There are three cases for the homogeneous solution, $u_{x}^{h}\left(x\right)$, to Equation (31) depending on the values of the system parameters $\lambda_{1}=k_{s}/{\left(EA\right)}_{}^{H}$ and $\lambda_{2}={\left(EA\right)}_{}^{L}/{\left(EA\right)}_{}^{H}$.

**Case I:** $\lambda_{2}<2\sqrt{\lambda_{1}}$

(33) $$\begin{array}{l} u_{x}^{h}\left(x\right)=C_{1}\cosh\left[\alpha x\right]\cos\left[\beta x\right]+C_{2}\sinh\left[\alpha x\right]\cos\left[\beta x\right]\\ \;\;\;\;\;\;\;\;+C_{3}\cosh\left[\alpha x\right]\sin\left[\beta x\right]+C_{4}\sinh\left[\alpha x\right]\sin\left[\beta x\right] \end{array}$$

**Case II:** $\lambda_{2}>2\sqrt{\lambda_{1}}$

(34) $$\begin{array}{l} u_{x}^{h}\left(x\right)=C_{1}\cosh\left[\alpha x\right]\cosh\left[\beta x\right]+C_{2}\sinh\left[\alpha x\right]\cosh\left[\beta x\right]\\ \;\;\;\;\;\;\;\;+C_{3}\cosh\left[\alpha x\right]\sinh\left[\beta x\right]+C_{4}\sinh\left[\alpha x\right]\sinh\left[\beta x\right] \end{array}$$

**Case III:**$\lambda_{2}=2\sqrt{\lambda_{1}}$

(35) $$u_{x}^{h}\left(x\right)=C_{1}e^{\sqrt[{4}]{\lambda_{1}}x}+C_{2}xe^{\sqrt[{4}]{\lambda_{1}}x}+C_{3}e^{-\sqrt[{4}]{\lambda_{1}}x}+C_{4}xe^{-\sqrt[{4}]{\lambda_{1}}x}$$

with the auxiliary variables α and β being defined as:

(36) $$\alpha =\sqrt{\frac{\sqrt{\lambda_{1}}}{2}+\frac{\lambda_{2}}{4}}$$

(37) $$\beta =\sqrt{\frac{\sqrt{\lambda_{1}}}{2}-\frac{\lambda_{2}}{4}}\;\;\mathrm{for}\;\;\mathrm{Case}\;\;I\;\;\;\;\mathrm{and}\;\;\beta =\sqrt{\frac{\lambda_{2}}{4}-\frac{\sqrt{\lambda_{1}}}{2}}\;\;\mathrm{for}\;\;\mathrm{Case}\;\;\mathrm{II}$$

where $C_{1}$, $C_{2}$, $C_{3}$, and $C_{4}$ are constants of integration to be obtained by enforcing boundary conditions.

### 5.4. Analytical Solution of the Nanobar–Substrate Medium System: Free Vibration Analysis

To obtain the analytical solution to the governing differential equation of motion of Equation (28), the method of separation of variables is utilized. Therefore, the axial displacement, $u_{x}\left(x,t\right)$, can be expressed as [59]:

(38) $$u_{x}\left(x,t\right)=\phi \left(x\right)e^{i\omega t}$$

where ω represents the natural frequency of the nanobar–substrate medium system.

Substituting Equation (38) into Equation (28) and setting $q_{x}\left(x,t\right)$ and P to be zero for the free vibration analysis leads to the following equation for the non-trivial solution ϕ(x):

(39) $$\frac{\partial^{4}\phi \left(x\right)}{\partial x^{4}}-\psi_{1}\frac{\partial^{2}\phi \left(x\right)}{\partial x^{2}}-\psi_{2}\phi \left(x\right)=0$$

where $\psi_{1}=\frac{{\left(EA\right)}^{L}}{{\left(EA\right)}^{H}}$ and $\psi_{2}=\frac{\rho A\omega^{2}-k_{s}}{{\left(EA\right)}^{H}}$ represent the auxiliary parameters.

As suggested by Dinçkal et al. [61], there are two possible solution cases for the non-trivial solution, ϕ(x):

**Case I:**$\rho A\omega^{2}-k_{s}>0$

(40) $$u\left(x\right)=C_{1}\cos\left[\beta_{1}x\right]+C_{2}\cosh\left[\beta_{2}x\right]+C_{3}\sin\left[\beta_{1}x\right]+C_{4}\sinh\left[\beta_{2}x\right]$$

with

(41) $$\beta_{1}=\frac{\sqrt{-\psi_{1}+\sqrt{\psi_{1}^{2}+4\psi_{2}}}}{\sqrt{2}};\;\;\beta_{2}=\frac{\sqrt{\psi_{1}+\sqrt{\psi_{1}^{2}+4\psi_{2}}}}{\sqrt{2}}$$

**Case II:**$\rho A\omega^{2}-k_{s}<0$

(42) $$\begin{array}{l} \phi \left(x\right)=C_{1}\cosh\left[\beta_{3}x\right]\cos\left[\beta_{4}x\right]+C_{2}\cosh\left[\beta_{3}x\right]\sin\left[\beta_{4}x\right]\\ \;\;\;\;\;\;\;+C_{3}\sinh\left[\beta_{3}x\right]\cos\left[\beta_{4}x\right]+C_{4}\sinh\left[\beta_{3}x\right]\;\sin\left[\beta_{4}x\right]\; \end{array}\;\mathrm{for}\;\psi_{1}<2\sqrt{\psi_{2}}$$

(43) $$\phi \left(x\right)=C_{1}\cosh\left[\beta_{5}x\right]+C_{2}\cosh\left[\beta_{6}x\right]+C_{3}\sinh\left[\beta_{5}x\right]+C_{4}\sinh\left[\beta_{6}x\right]\;\;\mathrm{for}\;\psi_{1}>2\sqrt{\psi_{2}}$$

(44) $$\phi \left(x\right)=C_{1}\cosh\left[\beta_{7}x\right]+C_{2}x\cosh\left[\beta_{7}x\right]+C_{3}\sinh\left[\beta_{7}x\right]+C_{4}x\sinh\left[\beta_{7}x\right]\;\;\mathrm{for}\;\psi_{1}=2\sqrt{\psi_{2}}$$

with

(45) $$\begin{array}{l} \beta_{3}=\sqrt{\frac{\sqrt{\psi_{2}}}{2}+\frac{\psi_{1}}{4}\;};\;\beta_{4}=\sqrt{\frac{\sqrt{\psi_{2}}}{2}-\frac{\psi_{1}}{4}\;};\beta_{5}=\frac{\sqrt{\psi_{1}+\sqrt{\psi_{1}^{2}-4\psi_{2}}}}{\sqrt{2}};\\ \beta_{6}=\frac{\sqrt{\psi_{1}-\sqrt{\psi_{1}^{2}-4\psi_{2}}}}{\sqrt{2}};\beta_{7}=\sqrt{\frac{\psi_{1}}{2}} \end{array}$$

## 6. Numerical Simulations

To assess both static and free vibration characteristics of SWCNT–substrate medium systems, this study employs the proposed nanobar–substrate medium model to analyze SWCNT–substrate medium systems through two numerical simulations. Each simulation contains two analysis cases and will be presented in the following sections.

### 6.1. Simulation I: Static Analysis

Simulation I investigates static responses of nanobar–substrate medium systems with two analysis cases. The first one considers only the material small-scale effect and demonstrates the ability of the proposed model to eliminate the paradoxical response inherent to the widely used small-scale bar model, while the second one assesses the influences of system parameters on the effective Young’s modulus of the nanobar–substrate system.

#### 6.1.1. Paradox Resolved

Several small-scale bar models available in the literature have shown no small-scale effect under a constant axial-force state, and this peculiar behavior has been considered as a paradox [62]. Therefore, the present analysis case is employed to show the ability of the proposed model to eliminate this paradoxical response.

**Figure 4:**
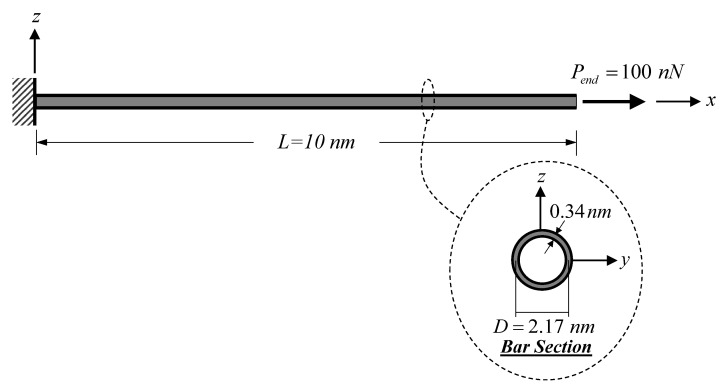
Cantilever SWCNT subjected to a constant axial-force state: static analysis.

Figure 4 shows a cantilever SWCNT subjected to an end load $P_{end}$ = 100 nN, thus inducing a constant axial-force state. As stated by Jena et al. [63], the bulk modulus, E, of the SWCNT is 1 TPa. The following geometric properties of the SWCNT follow those employed by Jena et al. [63]: length, L, of 10 nm, diameter, D, of 2.17 nm, and wall thickness, $t_{b}$, of 0.34 nm. To consider only the material small-scale effect, model parameters associated with the surface energy effect and the surrounding substrate medium are set to be zero ($E_{}^{sur}=\tau_{0}^{sur}=k_{s}=0$). The material length-scale parameters associated with the dilation gradient, $l_{0}$, and the deviatoric stretch gradient, $l_{1}$, are set to be identical ($l_{0}=l_{1}$) and vary from 0.5 to 2 nm. Three bar models based on different constitutive relations are used in this analysis case, namely: the proposed bar model, the local (classical) bar model, and the Eringen nonlocal bar model of Limkatanyu et al. [50]. As suggested by Akgöz and Civalek [59], imposed classical and non-classical boundary conditions for the cantilever SWCNT are:

(46) $${\;\;u_{x}\left(x\right)|}_{x=0}^{}=0\;\;\;\;\mathrm{and}\;{\left[-{\left(EA\right)}^{H}\frac{\partial^{3}u_{x}\left(x\right)}{\partial x^{3}}+{\left(EA\right)}^{L}\frac{\partial u_{x}\left(x\right)}{\partial x}\right]|}_{x=L}^{}=P_{end}\;$$

(47) $${{\left(EA\right)}^{H}\frac{\partial^{2}u_{x}\left(x\right)}{\partial x^{2}}|}_{x=0}^{}=0\;\mathrm{and}\;{\frac{\partial u_{x}\left(x\right)}{\partial x}|}_{x=L}^{}=0\;$$

**Figure 5:**
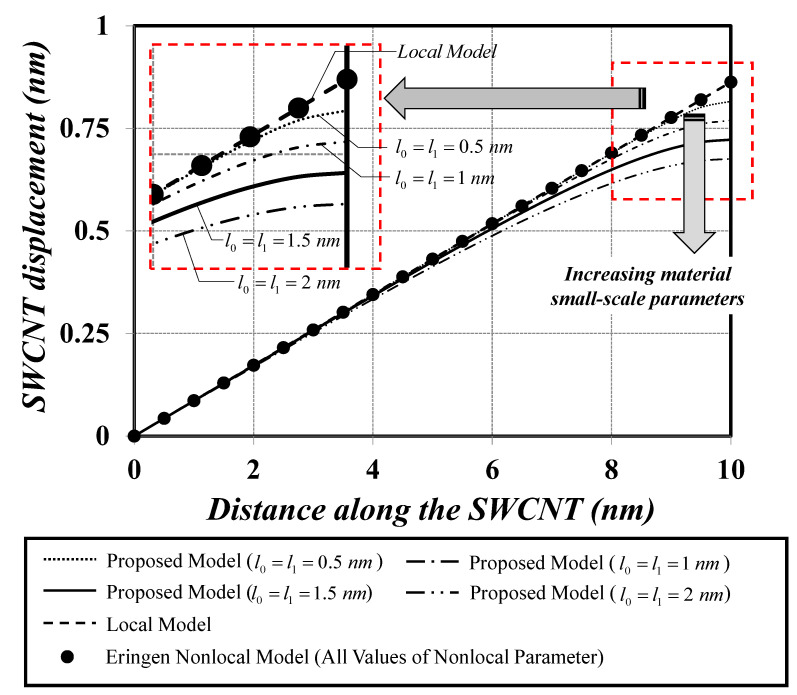
Axial displacement versus distance along the cantilever SWCNT under a constant axial-force state.

Figure 5 presents and compares the axial-displacement distributions obtained with all three bar models. Clearly, the axial-displacement response obtained with the Eringen nonlocal bar model is identical to that obtained with the local bar model. Therefore, the Eringen nonlocal differential model fails to represent the material small-scale effect under the constant axial-force state. Contrastingly, the modified strain-gradient bar model proposed herein succeeds in capturing the material small-scale effect and yields a stiffer axial-displacement response when compared to that obtained with the local bar model. This increased system stiffness complies well with experimental evidence and analytical results available in the literature [13,64,65].

#### 6.1.2. Parametric Investigation of System Parameters on the Effective Young’s Modulus

**Figure 6:**
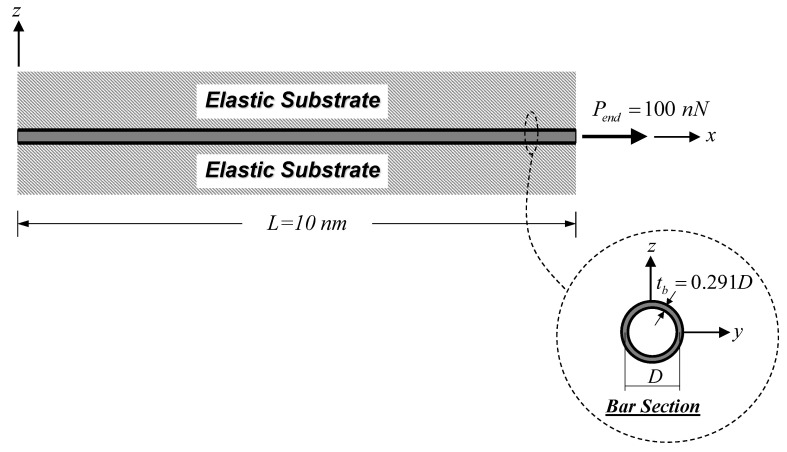
Free–free SWCNT–substrate medium system under an end force: static analysis.

Influences of several system parameters on the effective Young’s modulus, $E_{B}^{eff}$, are scrutinized in the present analysis case. A free–free SWCNT embedded in elastic substrate medium under an end load $P_{end}$ = 100 nN, as shown in Figure 6, is employed in this parametric investigation. The material and geometric properties of the SWCNT follow those employed in the previous analysis case. The surface property of the SWCNT is given by Jena et al. [63]. The elastic surface modulus, $E_{}^{sur}$, is 35.3 nN/nm, while the residual surface stress is assumed to be zero ($\tau_{0}^{sur}=0$). The material length-scale parameter associated with the deviatoric stretch gradient, $l_{1}$, is kept constant at 1 nm. System parameters assessed in this analysis case are the dilation-gradient small-scale parameter, $l_{0}$, the SWCNT diameter, D, and the elastic substrate stiffness, $k_{s}$. The material length-scale ratio, $l_{0}/l_{1}$, varies from 0 to 4. The slenderness ratio, L/D, ranging from 10 to 50 is employed to specify the SWCNT diameter parameter, thus reflecting the surface energy effect. A specified value of the slenderness ratio, L/D, can be obtained by keeping L = 10 nm and varying D. The corresponding wall thickness, $t_{b}$, of the SWCNT is equal to 0.291D, thus keeping the ratio $D/t_{b}$ constant at 3.44. This specific value of $D/t_{b}$ is given by Anvari [66]. The dimensionless substrate-stiffness parameter, ${\bar{K}}_{s}$, ranging from 1 to 100 is employed to vary the elastic substrate stiffness, $k_{s}$, and is defined as [50]:

(48) $${\bar{K}}_{s}=\frac{K_{s}\Gamma L^{2}}{{\left(EA\right)}_{}^{L}}$$

where $K_{s}$ represents a stiffness coefficient of the substrate medium and is defined as $K_{s}=k_{s}/D$. This range of the dimensionless substrate-stiffness parameter, ${\bar{K}}_{s}$, follows that employed by Demir et al. [67].

Classical and non-classical boundary conditions for the free–free SWCNT–substrate system follow those suggested by Akgöz and Civalek [59]:

(49) $$\begin{array}{ll} {\left[-{\left(EA\right)}^{H}\frac{\partial^{3}u_{x}\left(x\right)}{\partial x^{3}}+{\left(EA\right)}^{L}\frac{\partial u_{x}\left(x\right)}{\partial x}\right]|}_{x=0}^{}=0 & \mathrm{and}\;{\;\;\frac{\partial u_{x}\left(x\right)}{\partial x}|}_{x=0}^{}=0\;\;\\ \;{\left[-{\left(EA\right)}^{H}\frac{\partial^{3}u_{x}\left(x\right)}{\partial x^{3}}+{\left(EA\right)}^{L}\frac{\partial u_{x}\left(x\right)}{\partial x}\right]|}_{x=L}^{}=P_{end} & \mathrm{and}\;\;\;{\frac{\partial u_{x}\left(x\right)}{\partial x}|}_{x=L}^{}=0\;\;\; \end{array}$$

Employing the procedure suggested by He and Lilley [68], the effective Young’s modulus, $E_{B}^{eff}$, can be obtained in the following fashion: The end displacement, $u_{end}$, of the SWCNT–substrate medium system is first computed using the proposed model. Subsequently, Equation (50) is solved for $E_{B}^{eff}$.

(50) $$u_{end}=\frac{P_{end}\coth\left(L\sqrt{k_{s}/\left(E_{B}^{eff}A\right)}\right)}{\sqrt{k_{s}E_{B}^{eff}A}}$$

It is noted that the right-hand side of Equation (50) defines the end displacement analytically obtained from the local bar–substrate medium system.

**Figure 7:**
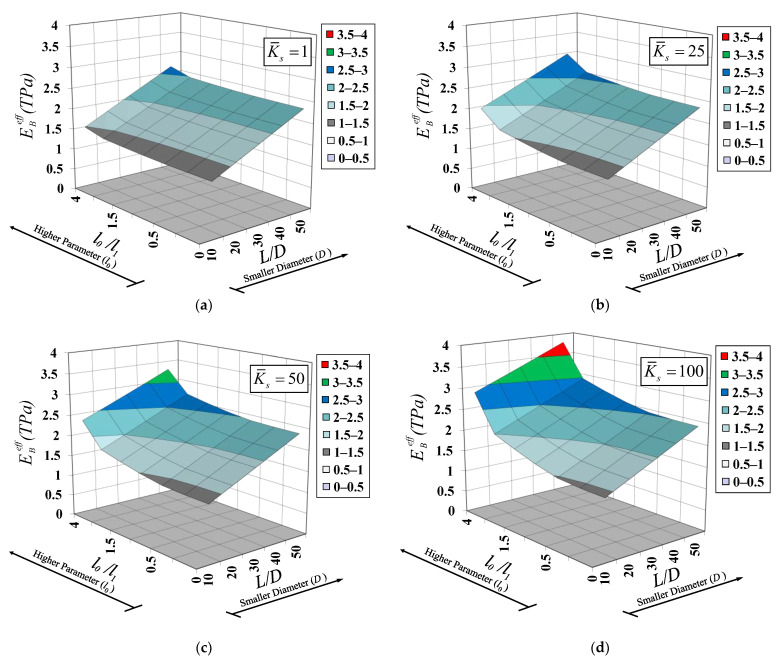
Variation of effective Young’s modulus with SWCNT diameter and material length-scale parameter for various elastic substrate stiffness: (**a**) ${\bar{K}}_{s}=1$, (**b**) ${\bar{K}}_{s}=25$, (**c**) ${\bar{K}}_{s}=50$, and (**d**) ${\bar{K}}_{s}=100$.

The variation of the effective Young’s modulus, $E_{B}^{eff}$, with all above-mentioned system parameters is shown Figure 7. It is worth mentioning that solution case I ($\lambda_{2}<2\sqrt{\lambda_{1}}$) of Equation (33) and solution case II ($\lambda_{2}>2\sqrt{\lambda_{1}}$) of Equation (34) are both activated in the present analysis case. Figure 7 shows that the effective Young’s modulus, $E_{B}^{eff}$, nonlinearly increases with increasing the dilation-gradient small-scale parameter, $l_{0}$, particularly for larger values of dimensionless substrate-stiffness parameters, ${\bar{K}}_{s}$ (stiff substrate media), and lower values of the SWCNT slenderness ratio, L/D (stubby SWCNT). This observation implies that the stiffening phenomenon associated with the dilatation gradient becomes more pronounced for a stiff substrate medium and a stubby SWCNT. It is worth pointing out that the stubby SWCNT results in lower values of the SWCNT surface area/bulk volume ratio, thus diminishing the stiffening effect associated with the surface-free energy [69]. With decreasing SWCNT diameters, D, the effective Young’s modulus, $E_{B}^{eff}$, increases in a linear fashion. Furthermore, it is observed that the variation of the effective Young’s modulus, $E_{B}^{eff}$, with the SWCNT diameters, D, is marginally affected by the dimensionless substrate-stiffness parameters, ${\bar{K}}_{s}$, for low values of the dilation-gradient small-scale parameter, $l_{0}$ ($l_{0}/l_{1}\leq 0.25$). The implication of this observation is that when the system stiffness enhancement is dominated by the surface-free energy, the variation of the effective Young’s modulus, $E_{B}^{eff}$, becomes invariant to the dimensionless substrate-stiffness parameters, ${\bar{K}}_{s}$. A similar observation was also noticed by Limkatanyu et al. [50].

### 6.2. Simulation II: Free Vibration Analysis

Simulation II focuses on free vibration responses of nanobar–substrate systems with two analysis cases. The first one examines the effects of material length-scale parameters and elastic substrate stiffness on the fundamental natural frequency, while the second one investigates the influences of the nanobar slenderness ratio (surface energy effect) and elastic substrate stiffness on natural frequencies for the first three vibration modes.

#### 6.2.1. Effect of Material Length-Scale Parameters on the Fundamental Natural Frequency of SWCNT–Substrate Medium Systems

**Figure 8:**
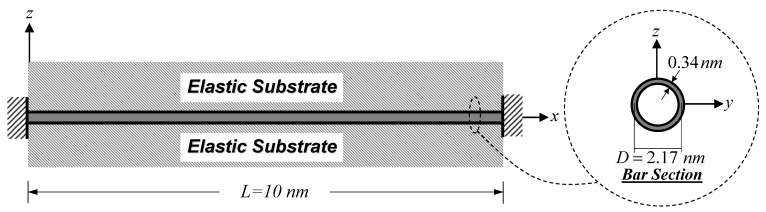
Clamped–clamped SWCNT embedded in the substrate medium: free vibration analysis.

In this analysis case, a clamped–clamped SWCNT embedded in the substrate medium shown in Figure 8 is employed to investigate the influence of material length-scale parameters on the natural frequency. The material and geometric properties as well as the surface modulus of the SWCNT follow those employed in Simulation I. The mass density of the SWCNT is 1,370 kg/m^3^, as given by Jena et al. [63]. Two non-dimensional parameters, $\xi_{0}=l_{0}/L$ and $\xi_{1}=l_{1}/L$, are employed to vary two material length-scale parameters associated with the dilation gradient, $l_{0}$, and the deviatoric stretch gradient, $l_{1}$, while the dimensionless substrate-stiffness parameter, ${\bar{K}}_{s}$, of Equation (48) is employed to vary the elastic substrate stiffness, $k_{s}$. The ranges of these parameters are $\xi_{0}=0-0.2$, $\xi_{1}=0-0.2$, and ${\bar{K}}_{s}=1-10$. The classical and non-classical boundary conditions of the clamped–clamped SWCNT follow those suggested by Akgöz and Civalek [59]:

(51) $$\begin{array}{ll} {u_{x}\left(0\right)|}_{x=0}^{}=0 & \mathrm{and}\;\;{{\left(EA\right)}^{H}\frac{\partial^{2}u_{x}\left(0\right)}{\partial x^{2}}|}_{x=0}=0\;\\ {u_{x}\left(L\right)|}_{x=L}^{}=0 & \mathrm{and}\;\;{{\left(EA\right)}^{H}\frac{\partial^{2}u_{x}\left(L\right)}{\partial x^{2}}|}_{x=L}=0\; \end{array}$$

Based on the properties of the SWCNT and the substrate medium employed in this analysis case, only the solution case I ($\rho A\omega^{2}-k_{s}>0$) of Equation (40) is activated. Substituting the non-trivial solution, ϕ(x), of Equation (40) into Equation (39) and enforcing the boundary conditions of Equation (51) yields the following matrix relation:

(52) $$\left[A\left(\omega \right)\right]C=\left[\begin{array}{cccc} 1 & 1 & 0 & 0\\ \cos\left(\beta_{1}L\right) & \cosh\left(\beta_{2}L\right) & \sin\left(\beta_{1}L\right) & \sinh\left(\beta_{2}L\right)\\ -\beta_{1}^{2} & \beta_{2}^{2} & 0 & 0\\ -\beta_{1}^{2}\cos\left(\beta_{1}L\right) & \beta_{2}^{2}\cosh\left(\beta_{2}L\right) & -\beta_{1}^{2}\sin\left(\beta_{1}L\right) & \beta_{2}^{2}\sinh\left(\beta_{2}L\right) \end{array}\right]\left[\begin{array}{c} C_{1}\\ C_{2}\\ C_{3}\\ C_{4} \end{array}\right]=0$$

The condition det[A(ω)]=0 yields the following characteristic equation:

(53) $$\sin\left(\beta_{1}L\right)=0\;\;\;\;\Rightarrow \;\;\;\beta_{1}=\frac{n\pi }{L}$$

Therefore, the explicit form of the natural frequency for the clamped–clamped SWCNT–substrate medium system can be obtained from Equation (53) as:

(54) $$\omega_{proposed}^{n}=\frac{\sqrt{L^{4}k_{s}+n^{2}\pi^{2}L^{2}{\left(EA\right)}^{L}+n^{4}\pi^{4}{\left(EA\right)}^{H}}}{\sqrt{\rho A}L^{2}}$$

with n being the vibration mode (n=1, 2, 3, …).

**Figure 9:**
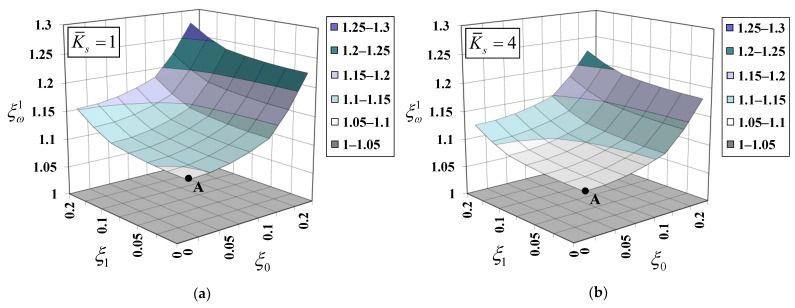

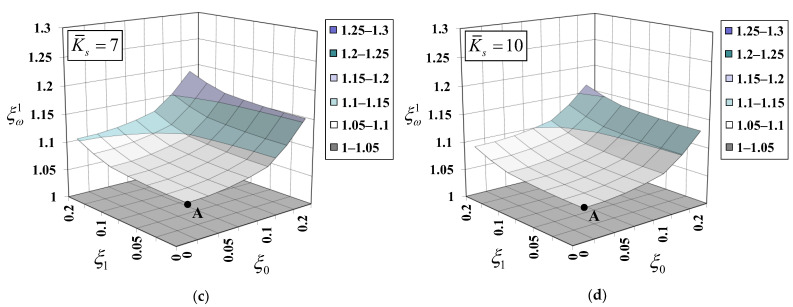
Variation of normalized natural frequency for the first vibration mode with material length-scale parameters for various elastic substrate stiffnesses: (**a**) ${\bar{K}}_{s}=1$, (**b**) ${\bar{K}}_{s}=4$, (**c**) ${\bar{K}}_{s}=7$, and (**d**) ${\bar{K}}_{s}=10$.

Figure 9 plots variations of the normalized natural frequency, $\xi_{\omega }^{1}=\omega_{proposed}^{1}/\omega_{classical}^{1}$, for the first vibration mode (n=1) with non-dimensional material length-scale parameters ($\xi_{0}$ and $\xi_{1}$) for various values of the dimensionless substrate-stiffness parameter, ${\bar{K}}_{s}$. The “*classical*” natural frequency, $\omega_{classical}^{1}$, for the first vibration mode is obtained with the bar–substrate medium model, in which material small-scale and surface energy effects are both excluded. Point A shown in Figure 9 represents the normalized natural frequency, $\xi_{\omega }^{1}$, when only the surface energy effect is considered ($\xi_{0}=\xi_{1}=0$). It is observed that the surface energy effect consistently enhances the system stiffness, thus increasing the natural frequency ($\xi_{\omega }^{1}>1.0$). However, this stiffening phenomenon is diminished by a stiffer substrate medium. The value of the normalized natural frequency, $\xi_{\omega }^{1}$, deceases from 1.098 to 1.056 when the value of the dimensionless substrate-stiffness parameter, ${\bar{K}}_{s}$, increases from 1 to 10. An increase in the normalized natural frequency, $\xi_{\omega }^{1}$, associated with the material small-scale effect is also noticed in Figure 9. Both non-dimensional material length-scale parameters ($\xi_{0}$ and $\xi_{1}$) enhance the system stiffness, but at different degrees of enhancement. The system stiffness enhancement associated with the dilation gradient, $l_{0}$, is more pronounced than that associated with the deviatoric stretch gradient, $l_{1}$. This observation was noticed by Akgöz and Civalek [59] as well. However, this system stiffening enhancement becomes less pronounced when a substrate medium becomes stiffer.

.

#### 6.2.2. Effect of Surface-Free Energy on Natural Frequencies of SWCNT–Substrate Medium Systems

**Figure 10:**
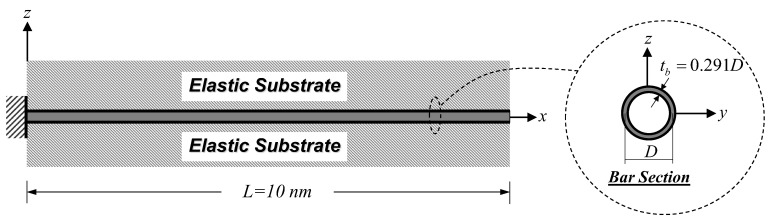
Cantilever SWCNT–substrate medium system: free vibration analysis.

To assess the surface energy effect on the system natural frequency, a clamped–free SWCNT embedded in the substrate medium shown in Figure 10 is employed in this analysis case. The material and geometric properties as well as the surface modulus of the SWCNT follow those employed in the previous analysis case. Two non-dimensional material length-scale parameters, $\xi_{0}=l_{0}/L$ and $\xi_{1}=l_{1}/L$, are kept constant at 0.1. System parameters assessed herein are the SWCNT slenderness ratio, L/D, and the elastic substrate stiffness, $k_{s}$. The slenderness ratio, L/D, is employed to specify the SWCNT diameter parameter and ranges from 5 to 50. A specified value of the slenderness ratio, L/D, can be obtained by keeping L = 10 nm and varying D. The corresponding wall thickness, $t_{b}$, of the SWCNT is equal to 0.291D, thus keeping the ratio $D/t_{b}$ constant at 3.44. This specific value of $D/t_{b}$ is given by Anvari [66]. The dimensionless substrate-stiffness parameter, ${\bar{K}}_{s}$, of Equation (48) is employed to vary the elastic substrate stiffness, $k_{s}$, and ranges from 1 to 10. The classical and non-classical boundary conditions follow those suggested by Akgöz and Civalek [59], as presented in Equations (46) and (47). Following the properties of the SWCNT and substrate medium employed in this analysis case, the solution case I ($\rho A\omega^{2}-k_{s}>0$) of Equation (40) is activated. Substituting the non-trivial solution, ϕ(x), of Equation (40) into Equation (39) and enforcing the boundary conditions of Equations (46) and (47) yields the following matrix relation:

(55) $$\left[A\left(\omega \right)\right]C=\left[\begin{array}{cccc} 1 & 1 & 0 & 0\\ -\sin\left(\beta_{1}L\right) & \frac{\Phi_{2}}{\Phi_{1}}\sinh\left(\beta_{2}L\right) & \cos\left(\beta_{1}L\right) & \frac{\Phi_{2}}{\Phi_{1}}\cosh\left(\beta_{2}L\right)\\ -\beta_{1}^{2} & \beta_{2}^{2} & 0 & 0\\ -\sin\left(\beta_{1}L\right) & \frac{\beta_{2}^{}}{\beta_{1}^{}}\sinh\left(\beta_{2}L\right) & \cos\left(\beta_{1}L\right) & \frac{\beta_{2}^{}}{\beta_{1}^{}}\cosh\left(\beta_{2}L\right) \end{array}\right]\left[\begin{array}{c} C_{1}\\ C_{2}\\ C_{3}\\ C_{4} \end{array}\right]=0$$

where $\Phi_{1}={\left(EA\right)}^{L}\beta_{1}+{\left(EA\right)}^{H}\beta_{1}^{3}$ and $\Phi_{2}={\left(EA\right)}^{L}\beta_{1}-{\left(EA\right)}^{H}\beta_{1}^{3}$.

The condition det[A(ω)]=0 yields the following characteristic equation:

(56) $$\cos\left(\beta_{1}L\right)=0\;\;\;\;\Rightarrow \;\;\;\beta_{1}=\frac{\left(2n-1\right)\pi }{2L}$$

Therefore, the explicit form of the natural frequency for the clamped–free SWCNT–substrate medium system can be obtained from Equation (56) as:

(57) $$\omega_{proposed}^{n}=\frac{\sqrt{16L^{4}k_{s}+4{\left(1-2n\right)}^{2}\pi^{2}L^{2}{\left(EA\right)}^{L}+{\left(1-2n\right)}^{4}\pi^{4}{\left(EA\right)}^{H}}}{4\sqrt{\rho A}L^{2}}$$

with n being the vibration mode (n=1, 2, 3, …).

**Figure 11:**
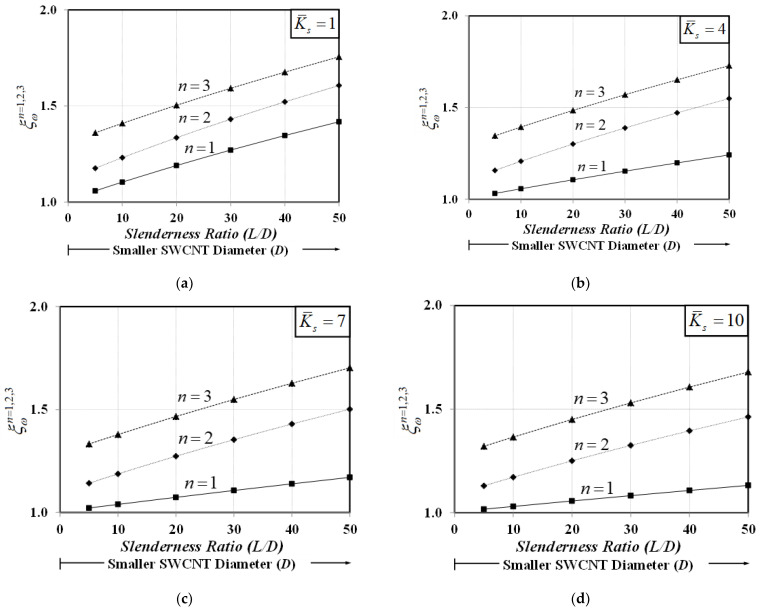
Variation of normalized natural frequencies for the first three vibration modes with SWCNT diameter for various elastic substrate stiffnesses: (**a**) ${\bar{K}}_{s}=1$, (**b**) ${\bar{K}}_{s}=4$, (**c**) ${\bar{K}}_{s}=7$, and (**d**) ${\bar{K}}_{s}=10$.

Figure 11 plots variations of the normalized natural frequency, $\xi_{\omega }^{n}=\omega_{proposed}^{n}/\omega_{classical}^{n}$, for the first three vibration modes (n=1−3) with the SWCNT slenderness ratio, L/D, for various values of the dimensionless substrate-stiffness parameter, ${\bar{K}}_{s}$. The “*classical*” natural frequency, $\omega_{classical}^{n}$, is obtained with the bar–substrate medium model, in which material small-scale and surface energy effects are both excluded. As pointed out by Ponbunyanon et al. [69], the bar surface area/bulk volume ratio increases with the increasing bar slenderness ratio, thus rendering the surface energy effect more pronounced. Figure 11 shows that the normalized natural frequencies $\xi_{\omega }^{1}$, $\xi_{\omega }^{2}$, and $\xi_{\omega }^{3}$ increase with the increasing slenderness ratio, L/D (decreasing diameter, D), especially for higher vibration modes. This observation implies that the stiffening phenomenon induced by the surface energy magnifies all system natural frequencies and is in good agreement with that of Ebrahimi et al. [70]. However, this system stiffening enhancement becomes less pronounced when a substrate medium becomes stiffer, especially for the first vibration mode. In other words, the size-dependency induced by the surface-free energy is stabilized by a stiff substrate medium.

## 7. Conclusions

The present work proposed a novel bar–substrate medium model with the inclusion of material small-scale and surface energy effects for both static and free vibration analyses of SWCNT–substrate medium systems. The material small-scale effect of the SWCNT was represented by the modified strain-gradient theory, while the surface energy effect was considered by the Gurtin–Murdoch surface model. The Winkler foundation model was employed to account for the interactive mechanism between the SWCNT and its surrounding substrate medium. Within the framework of Hamilton’s principle, the system equation of motion as well as associated initial and boundary conditions were derived in a consistent manner. To show the essence of the material small-scale effect, the surface energy effect, and the surrounding substrate medium on static and free vibration responses of SWCNT–substrate medium systems, two numerical simulations were employed.

The first numerical simulation collected two analysis cases and focused on static responses of SWCNT–substrate medium systems. The first analysis case showed that the modified strain-gradient bar model was able to represent the material small-scale effect under the constant axial-force state, thus eliminating the famous paradoxical characteristic of the Eringen nonlocal bar model. The material small-scale effect accounted for by the modified strain-gradient theory led to a system-stiffness enhancement. The second analysis case showed that the effective Young’s modulus, $E_{B}^{eff}$, of the SWCNT–substrate medium system was enlarged upon increasing the dilation-gradient small-scale parameter, $l_{0}$, especially for large values of dimensionless substrate-stiffness parameters, ${\bar{K}}_{s}$ (stiff substrate media), and lower values of the SWCNT slenderness ratio, L/D (stubby SWCNT).

The second numerical simulation contained two analysis cases and emphasized on free vibration responses of SWCNT–substrate medium systems. The first analysis case demonstrated that the normalized fundamental frequency of the SWCNT–substrate medium system increased with the increasing non-dimensional material length-scale parameters, particularly for low values of dimensionless substrate-stiffness parameters, ${\bar{K}}_{s}$ (soft substrate media). However, the frequency enhancement associated with the dilation gradient, $l_{0}$, was more pronounced than that associated with the deviatoric stretch gradient, $l_{1}$. The second analysis case showed that the normalized natural frequencies $\xi_{\omega }^{1}$, $\xi_{\omega }^{2}$, and $\xi_{\omega }^{3}$ increased with the increasing slenderness ratio, L/D (more pronounced surface energy effect), especially for higher vibration modes. However, this system frequency enhancement became less noticeable when the substrate medium became stiffer, especially for the first vibration mode.

## Data Availability

Data are available upon reasonable request from the corresponding author.
